# Factors associated with falls among hospitalized and community-dwelling older adults: the APPCARE study

**DOI:** 10.3389/fpubh.2023.1180914

**Published:** 2023-06-29

**Authors:** Esmée L. S. Bally, Lizhen Ye, Amy van Grieken, Siok Swan Tan, Francesco Mattace-Raso, Elena Procaccini, Tamara Alhambra-Borrás, Hein Raat

**Affiliations:** ^1^Department of Public Health, Erasmus MC University Medical Center, Rotterdam, Netherlands; ^2^Research Group City Dynamics, InHolland University of Applied Sciences, Rotterdam, Netherlands; ^3^Division of Geriatric Medicine, Erasmus MC University Medical Center, Rotterdam, Netherlands; ^4^Funded Project Office, Local Health Authority n.2 Treviso, Treviso, Italy; ^5^Polibienestar Research Institute, University of Valencia, Valencia, Spain

**Keywords:** accidental falls, risk factors, older adults, aging, prevention

## Abstract

**Background:**

Falls are a leading cause of disability. Previous studies have identified various risk factors for falls. However, contemporary novel research is needed to explore these and other factors associated with falls among a diverse older adult population. This study aims to identify the factors associated with falls among hospitalized and community-dwelling older adults.

**Methods:**

Cross-sectional data from the ‘Appropriate care paths for frail elderly people: a comprehensive model’ (APPCARE) study were analyzed. The study sample consisted of hospitalized and community-dwelling older adults. Falling was assessed by asking whether the participant had fallen within the last 12 months. Multivariable logistic regression models were used to evaluate associations between socio-demographic characteristics, potential fall risk factors and falls.

**Results:**

The sample included 113 hospitalized (mean age = 84.2 years; 58% female) and 777 community-dwelling (mean age = 77.8 years; 49% female) older adults. Among hospitalized older adults, loneliness was associated with an increased risk of falls. Associations between female sex, secondary education lever or lower, multimorbidity, a higher score on limitations with activities of daily living (ADL), high risk of malnutrition and falling were found among community-dwelling participants.

**Conclusion:**

The results of this study confirm the multi-factorial nature of falling and the complex interaction of risk factors. Future fall prevention programs could be tailored to the needs of vulnerable subpopulations at high risk for falls.

## 1. Introduction

A fall can be defined as “an unexpected event in which the participant comes to rest on the ground, floor, or lower level” (1). One in three community-dwelling older adults fall each year (2), and the incidence rate of fall-related injuries increases with age (3). After a fall, 20 to 30% of older adults have moderate to severe injuries (1). In approximately 10% of all cases falls can lead to serious injuries that require hospitalization, such as fractures, joint dislocations, and head injury (2). Falling affects not only the health of older adults, but also places a high burden on public health resources (4). In 2021, in the Netherlands, the estimated direct medical costs were 1.5 billion euros (5) which is expected to increase to 2.7 billion by 2040 as the population ages (6).

Falls can have multiple causes resulting from the complex interaction of risk factors. Previous studies have identified several predicting factors for falls in the general population, including socio-demographic (e.g., age, sex), biological (e.g., history of falls, visual impairment), environmental (e.g., home hazards, physical disability), behavioral (e.g., medication intake, sedentary behavior) and cognitive-related factors (7–10). Among community-dwelling older adults, previous studies identified older age (7-10), being female (7, 10), the presence of multiple health conditions (9), being frail (7), having mobility limitations (8–10) using multiple medications (7, 9) and having depressive symptoms (7, 8) as the predictors most strongly associated with falls.

However, differences may exist between hospitalized and community-dwelling older adults. Identifying the factors associated with falls among subpopulations is crucial in fall prevention (11). It enables the identification of older adults who are at high risk of falling, thereby allowing tailored fall prevention programs for various groups at risk. The aim of the current study is to identify the factors associated with falls among hospitalized and community-dwelling older adults. The factors included in the study are socio-demographic characteristics and potential fall risk factors (e.g., health indicators, lifestyle factors).

## 2. Methods

### 2.1. Study design

A cross-sectional study was performed using data from the ‘Appropriate care paths for frail elderly people: a comprehensive model’ (APPCARE) study. APPCARE is a prospective cohort study funded by the European Commission, under Grant Agreement No. 664689. APPCARE aimed to promote healthy aging. The project has been conducted in three European sites (Rotterdam, the Netherlands; Treviso, Italy, and; Valencia, Spain). The current study used baseline data from the Rotterdam site.

### 2.2. Participants

The study sample consisted of hospitalized and community-dwelling older adults. Three hospitals located in the Rotterdam region contributed to the recruitment of hospitalized older adults. All patients ≥70 years and older that entered the geriatric ward were invited by their healthcare provider to participate in the study, resulting in a total of 137 invited patients. Additionally, in collaboration with the Municipality of Rotterdam, 865 community-dwelling older adults (≥65 years) were invited by letter. An information package with an information sheet, informed consent form, baseline questionnaire, and prepaid envelope was distributed by post. Data were collected between 2017 and 2018. Participants who provided informed consent and completed the baseline questionnaire were included in the study. The Medical Ethics Committee of Erasmus MC University Medical Center in Rotterdam declared that the rules laid down in the Medical Research Involving Human Subjects Act (also known by its Dutch abbreviation WMO), do not apply to this research (reference number: MEC-2016-559).

**Figure 1:**
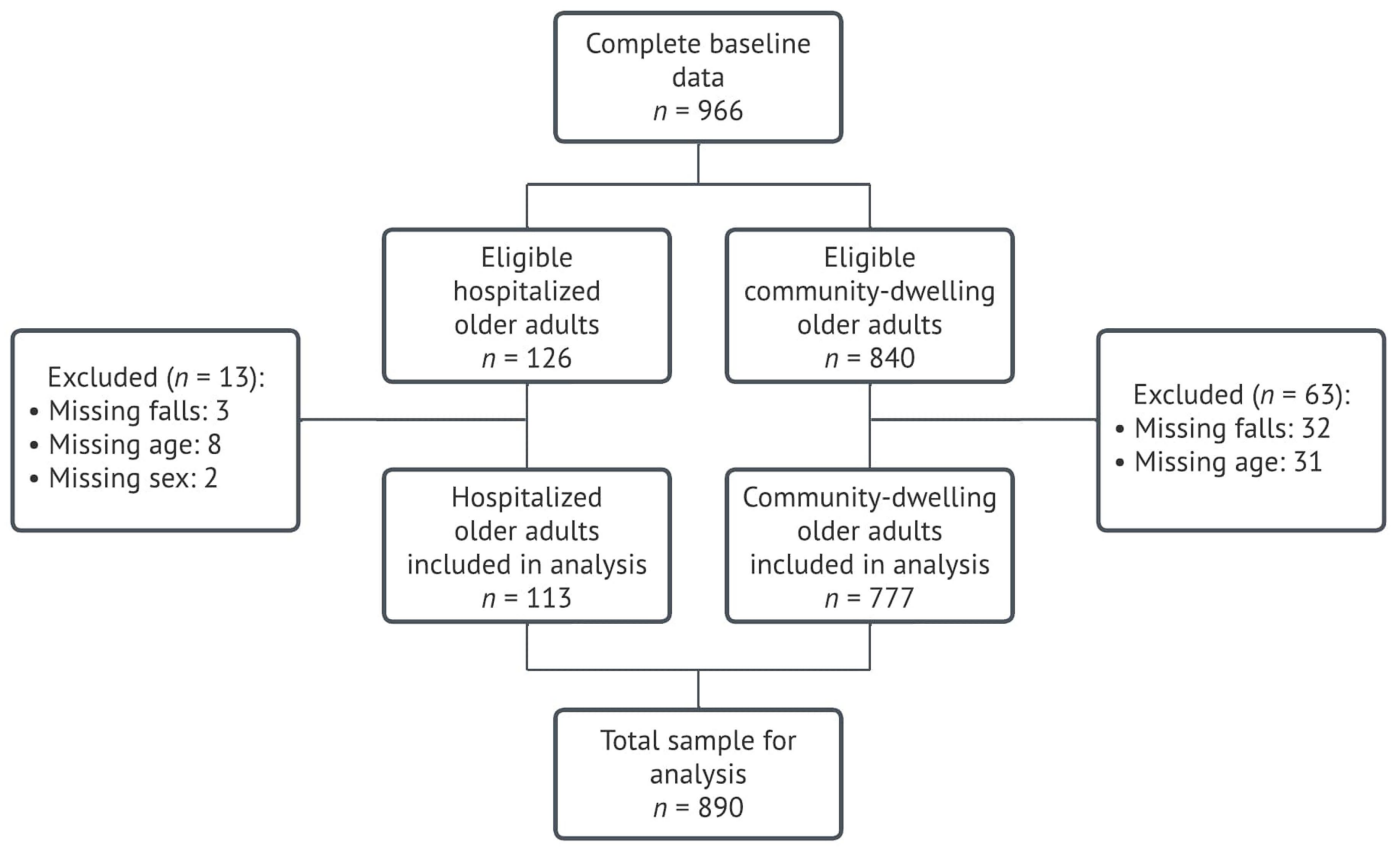
Population of analysis.

Data from 966 participants who provided informed consent and filled in the baseline questionnaire were available for this study. In order to conduct the full analysis, participants with missing data in the outcome variable (*n* = 35), age (*n* = 39), and sex (*n* = 2) were excluded, resulting in 890 (92.1%) subjects included. A flow diagram of the population of analysis is presented in Figure 1.

### 2.3. Measures

#### 2.3.1. Falling

The outcome measure used in this study is rate of falling. Falls were self-reported and assessed by asking participants “Have you had a fall in the last 12 months?” (12). Fall status was dichotomized into has fallen one or more times versus no falls.

#### 2.3.2. Socio-demographic factors

Socio-demographic characteristics were assessed at baseline and included as covariates. Age (in years) was provided by participants and categorized into 65–79 years and ≥ 80 years for logistic regression. This cut-off was chosen based on publications by the World Health Organization, in which the oldest-old is defined as people aged 80 or older (2, 13). Living situation was categorized into living with others or living alone. Education level concerned the highest level of education the participant achieved and was split into two categories based on the International Standard Classification of Education (ISCED). ISCED level 0–5 was categorized as ‘secondary or lower’ and ISCED level 6–8 was categorized as ‘tertiary or higher’ (14).

#### 2.3.3. Fall risk factors

Variables reported as risk factors in the literature (15, 16) and assessed at baseline were considered as associated factors: multimorbidity, frailty, limitations with activities of daily living (ADL), loneliness, risk of medication-related problems, risk of malnutrition, physical activity and poor vision. Multimorbidity was defined as having two or more medical conditions and/or disabilities at the same time (17). Participants were asked whether they had one or more chronic conditions diagnosed by a medical professional. A list of 13 common chronic conditions (e.g., high blood pressure, stroke, diabetes) was provided to participants to select from (18). Participants could add any health condition that was not listed. Frailty was assessed by the 15-item Tilburg Frailty Indicator (TFI) (19). The score on overall frailty ranged from 0 to 15. Participants with a total TFI-score ≥ 5 were considered frail (19). ADL limitations were measured with the Groningen Activity Restriction Scale (GARS) (20). Response categories by activities were: ‘Yes, I can do this fully independently, without any difficulty’, ‘Yes, I can do this fully independently, but with some difficulty’, ‘Yes, I can do this fully independently, but with great difficulty’, and ‘No, I can only do it with someone’s help’. Answers were assessed on a 4-point scale with a minimum score of 18 and maximum score of 72. Higher scores represented a lower level of independence. Loneliness was measured using the 6-item De Jong-Gierveld Loneliness Scale (21). Answer options were ‘no’, ‘more or less’ and ‘yes’. Scores were calculated according to the guidelines (22). Scores on overall loneliness varied between 0 or 1 ‘No loneliness’, 2 to 4 ‘Moderately intense loneliness’, and 5 or 6 ‘Intense loneliness’. In this study, the total loneliness score was dichotomized in ‘not lonely’ (score 0 or 1) and ‘lonely’ (score 2 through 6). Risk of medication-related problems was evaluated by the Medication Risk Questionnaire (MRQ) (23). The MRQ includes questions to assess polypharmacy, inappropriate prescribing, and poor adherence. The sum of eight items of the MRQ was used to calculate the risk of medication-related problems (23). Participants were classified as ‘low risk’ (score 0 through 3) or ‘high risk’ (score 4 or higher) (24). Risk of malnutrition was measured following the guidelines of the Short Nutritional Assessment Questionnaire 65+ (SNAQ65+) (25) and dichotomized in ‘low risk’ and ‘high risk’. SNAQ65+ consists of 4 questions: mid-upper arm circumference (<25 cm), unintentional weight loss (≥4 kg last six months), appetite, and walking stairs. As this study used self-reported data, an assessment of mid-upper-arm circumference was not available. Therefore, mid-upper arm circumference was excluded from score calculation. Instead, more emphasis was placed on unintentional weight loss which was measured by one item of the TFI (26). If a participant lost 6 kg or more during the last 6 months, or 3 kg or more during the last month this was categorized as high risk of malnutrition. Participants with poor appetite and problems with walking stairs and no weight loss, or no indications at all for malnutrition, were categorized as low risk. Physical activity was measured by one item of The Frailty Instrument of the Survey of Health, Aging and Retirement in Europe (SHARE-FI) (27). Participants were asked to indicate the frequency of activities requiring low to medium energy levels, such as gardening or going for a walk. Answers were dichotomized into ‘once a week or less’ or ‘more than once a week’. Poor vision was assessed by asking participants ‘Do you experience problems in your daily life due to poor vision?’ If participants answered ‘yes’ to this question, this was categorized as poor vision.

### 2.4. Statistical analyses

The analyses were done separately for the two sub-samples (hospitalized and community-dwelling older adults). Analyses were conducted using SPSS version 25.0 (IBM Corp., Armonk, NY, United States). Participant characteristics were analyzed using descriptive statistics. Characteristics of fallers and non-fallers were compared by *t*-test for continuous variables and by means of chi-square tests for categorical variables. Multivariable logistic regression was used to assess associations between the associated factors and falling. Odds ratio’s (OR) with 95% confidence intervals (95% CI) were calculated for each factor. Results were considered significant at *p <* 0.05. To evaluate whether the effect of associated factors on falling was modified by socio-demographic factors (age, sex, education level, household composition), an interaction term was added to the model. The interaction term socio-demographic factor*variable was tested in the multivariate logistic model, for each variable separately and with adjustment for all the other variables. Subsequently, Bonferroni correction for multivariable logistic regression was applied for analysis of the interaction items (*p* = 0.05/38 = 0.001) (28).

## 3. Results

### 3.1. Participant characteristics

Table 1 presents the socio-demographic and fall-risk characteristics of hospitalized (*n* = 113) and community-dwelling (*n* = 777) older adults at baseline. The mean age of hospitalized and community-dwelling older adults was 84.2 years ± 6.8 years and 77.8 years ± 6.3 years, respectively. Among hospitalized older adults, 57.5% were women compared to 48.9% among community-dwelling older adults. A total of 72 (63.7%) hospitalized and 213 (27.4%) community-dwelling older adults reported at least one fall within the past 12 months, with an overall mean of 32%.

**Table 1:** Baseline characteristics of hospitalized older adults (*n* = 113) and community-dwelling older adults (*n* = 777).

	Hospitalized older adults				Community-dwelling older adults			
	Total (*n* = 113)	Falls			Total (*n* = 777)	Falls		
		No (*n* = 41)	Yes (*n* = 72)	*p*-value		No (*n* = 564)	Yes (*n* = 213)	*p*-value
Age (years)	84.2 ± 6.8	83.1 ± 7.3	84.9 ± 6.4	0.218^a^	77.8 ± 6.3	77.0 ± 5.9	79.9 ± 7.0	**<0.001** ^**a**^
Sex, female	65 (57.5%)	22 (53.7%)	43 (59.7%)	0.531^b^	380 (48.9%)	258 (45.7%)	122 (57.3%)	**0.004** ^**b**^
Education level								
Secondary or lower	88 (83.8%)	31 (81.6%)	57 (85.1%)	0.640^b^	614 (80.2%)	447 (80.5%)	167 (79.1%)	0.666^b^
Tertiary or higher	17 (16.2%)	7 (18.4%)	10 (14.9%)		152 (19.8%)	108 (19.5%)	44 (20.9%)	
Household composition, living alone	70 (67.3%)	27 (69.2%)	43 (66.2%)	0.746^b^	314 (42.1%)	205 (37.6%)	109 (54.2%)	**<0.001** ^**b**^
Multimorbidity, yes	96 (86.5%)	33 (84.6%)	63 (87.5%)	0.671^b^	586 (75.9%)	400 (71.2%)	186 (88.6%)	**<0.001** ^**b**^
Frailty, yes	82 (75.9%)	27 (71.1%)	55 (78.6%)	0.383^b^	239 (32.6%)	135 (25.0%)	104 (53.9%)	**<0.001** ^**b**^
ADL (GARS; score)	41.4 ± 14.1	37.4 ± 14.2	43.0 ± 13.8	0.737^a^	24.9 ± 9.9	23.0 ± 8.2	29.9 ± 12.0	**<0.001** ^**a**^
Loneliness, yes	58 (54.7%)	17 (45.9%)	41 (59.4%)	0.184^b^	296 (39.3%)	187 (34.2%)	109 (52.7%)	**<0.001** ^**b**^
Risk of medication-related problems, yes	58 (54.2%)	14 (37.8%)	44 (62.9%)	**0.013** ^**b**^	268 (34.8%)	167 (29.9%)	101 (47.9%)	**<0.001** ^**b**^
Risk of malnutrition, yes	48 (42.5%)	18 (43.9%)	30 (41.7%)	0.817^b^	53 (6.8%)	17 (3.0%)	36 (16.9%)	**<0.001** ^**b**^
Physical activity								
Once a week or less	71 (68.3%)	22 (62.9%)	49 (71.0%)		220 (28.6%)	131 (23.4%)	89 (43.0%)	**<0.001** ^**b**^
More than once a week	33 (31.7%)	13 (37.1%)	20 (29.0%)	0.398^b^	548 (71.4%)	430 (76.6%)	118 (57.0%)	
Poor vision, yes	30 (27.0%)	12 (29.3%)	18 (25.7%)	0.684^b^	122 (16.9%)	74 (13.9%)	48 (25.3%)	**<0.001** ^**b**^

SD, standard deviation; ADL, Activities of Daily Living; GARS, Groningen Activities Restriction Scale. Presented as mean ± SD or N (%); Significant p-values (< 0.05) in bold. Missing items hospitalized sample: Education level = 8; Household composition = 9; Multimorbidity = 2; Frailty = 5; ADL = 5; Loneliness = 7; Medication risk = 6 Physical activity = 9; Vision = 2. Missing items community-dwelling sample: Education level = 11; Household composition = 31; Multimorbidity = 5; Frailty = 43; ADL = 9; Loneliness = 23; Medication risk = 7 Physical activity = 9; Vision = 53.

a *p*-values based on independent *t* test.

b *p*-values based on chi-square test.

In the hospital group, fallers were at higher risk of medication-related problems (*p* = 0.013), compared to non-fallers. Among community-dwelling participants, fallers were older (*p* < 0.001), more often women (*p* = 0.004), lived alone more frequently (*p* < 0.001) and had more often multimorbidity (*p* < 0.001), compared to non-fallers. In addition, participants who experienced a fall were at higher risk of frailty, loneliness, medication-related problems and malnutrition (*p* < 0.001), compared to participants who did not fall. Furthermore, community-dwelling participants who fell were less likely to engage in physical activity more than once a week and more subject to ADL limitations and poor vision (*p* < 0.001).

### 3.2. Factors associated with falling

The results of the multivariate logistic regression models for falling per group are presented in Table 2. When controlling for all factors in the model, hospitalized participants who were classified as lonely had 3.04 (95% CI: 1.08–8.57) times higher odds of falling compared to participants who were not at risk of loneliness. There were no other significant associations between potential associated factors and falling among participants who were admitted to the hospital.

**Table 2:** Multivariate logistic regression models on associations between associated factors and falls of hospitalized older adults (*n* = 113) and community-dwelling older adults (*n* = 777).

	Hospitalized older adults		Community-dwelling older adults	
	*n*=113^a^		*n*=777^b^	
	OR (95% CI)	*p*-value	OR (95% CI)	*p*-value
Age				
65–79 years	Ref		Ref	
≥80 years	2.89 (0.75–11.20)	0.124	1.21 (0.79–1.85)	0.374
Sex				
Male	Ref		Ref	
Female	1.10 (0.36–3.34)	0.873	**1.57 (1.05–2.36)**	**0.029**
Education level				
Tertiary or higher	Ref		Ref	
Secondary or lower	1.51 (0.37–6.11)	0.566	**0.47 (0.30–0.75)**	**0.002**
Household composition				
Living with others	Ref		Ref	
Living alone	0.79 (0.25–2.54)	0.694	1.02 (0.66–1.56)	0.945
Multimorbidity				
0–1 health conditions	Ref		Ref	
≥2 health conditions	0.69 (0.09–5.21)	0.716	**2.07 (1.14–3.77)**	**0.017**
Frailty status				
Not frail	Ref		Ref	
Frail	1.28 (0.32–5.13)	0.730	1.42 (0.83–2.44)	0.200
ADL (GARS; score)	1.01 (0.97–1.05)	0.584	**1.03 (1.00–1.06)**	**0.025**
Loneliness				
Not lonely	Ref		Ref	
Lonely	**3.04 (1.08–8.57)**	**0.036**	1.07 (0.70–1.64)	0.762
Medication-related problems				
Low risk	Ref		Ref	
High risk	2.34 (0.82–6.72)	0.113	1.39 (0.92–2.10)	0.113
Malnutrition				
Low risk	Ref		Ref	
High risk	0.76 (0.27–2.12)	0.602	**3.05 (1.45–6.42)**	**0.003**
Physical activity				
More than once a week	Ref		Ref	
Once a week or less	1.34 (0.43–4.18)	0.612	1.51 (0.96–2.38)	0.076
Vision				
Sufficient vision	Ref		Ref	
Poor vision	0.64 (0.20–2.07)	0.452	1.06 (0.63–1.78)	0.823

OR, odds ratio; CI, confidence interval; ADL, Activities of Daily Living; GARS, Groningen Activities Restriction Scale. Significant ORs and *p*-values (< 0.05) in bold. Multivariate logistic regression was used to analyze the association between potential associated factors and falls. All associated factors were included in one model.

a Nagelkerke *R* square = 0.18.

b Nagelkerke *R* square = 0.20.

For community-dwelling participants, the multivariate regression model showed that female sex (OR = 1.57, 95% CI: 1.05–2.36) was associated with higher odds of falling. Older adults with a secondary education level or lower (OR = 0.47, 95% CI: 0.30–0.75) were at lower risk of falling compared to older adults tertiary education level or higher. In addition, participants with multimorbidity were at higher risk of falling (OR = 2.07, 95% CI: 1.14–3.77) compared to participants with less than two health conditions. Having a higher score on ADL limitations was also significantly associated with falling (OR = 1.03, 95% CI: 1.00–1.06). Finally, older adults at high risk of malnutrition had a 3.05 (95% CI: 1.45–6.42) times higher odds of falling compared to participants who were at low risk of malnutrition. Interaction analyses revealed no statistically significant interactions after Bonferroni correction was applied.

## 4. Discussion

This study aimed to identify the factors associated with falls among hospitalized and community-dwelling older adults. In our sample, falling was associated with female sex, education level, multimorbidity, a higher score on ADL limitations, loneliness and a high risk of malnutrition.

With respect to hospitalized older adults, participants who were indicated as lonely were more prone to falling compared to participants who were not at risk of loneliness. The direct relationship between loneliness and falls remains unclear. However, results of previous studies indicated that social isolation, living alone and low social contact were associated with falls (29–31). A possible explanation is that social relationships can result in increased access to health care services and medication compliance, reducing the risk of falling (30). Other studies suggest a link between feelings of loneliness, depression and falls (29, 32). Symptoms of major depression, such as psychomotor retardation, slow gait speed and low energy can lead to falls (32). Further research is needed to explore whether older adults are depressed due to feelings of loneliness. There were no other significant associations between potential associated factors and falling among hospitalized participants. Further research in a larger sample size is needed to examine what other factors are associated with falls in hospitalized older adults.

Among community-dwelling older adults, women were more likely than men to fall. Previous studies have reported gender differences in falls in which women are disproportionally affected (33, 34). A possible explanation is that women’s bone mass decline faster than that of men, especially following menopause. This affects their physical functioning, thus increasing the risk of falling (33). Remarkably, participants who completed secondary education or lower had a relatively lower risk of falling compared to participants with a higher education level. This was not reported in the literature. In general, older adults with higher education levels have more confidence in their ability to avoid falling (35). Further studies are recommended to gain more insight in the association between education level and falling.

The association between multimorbidity and falling among community-dwelling adults is consistent with previous findings (36, 37). Sibley et al. observed a linear trend between the number of chronic diseases and fall rate (37). More specifically, age-related health conditions such as neurodegenerative diseases were found to be major risk factors for falls (38). Aging causes loss of muscle mass and muscular strength which could lead to loss of balance and coordination resulting in falls (39).

Mobility and balance problems have been shown to be most important in the etiology of falling (39, 40). Among community-dwelling older adults, fallers had a significant higher score on ADL limitations compared to non-fallers in this group. ADL limitations could lead to slow gait speed and impact balance increasing the risk of falls (40). These findings are concurrent to the findings of other studies (8, 40, 41).

Furthermore, the present study confirms a high risk of malnutrition to be a predictor of falls in community-dwelling older adults. This is in line with findings of previous studies (42, 43). Deficiencies in nutrients can result in low body mass index which is associated with a higher risk of falls. Malnutrition in older adults is correlated with co-morbidities such as sarcopenia and frailty, increasing the risk of falling by reduced muscle strength, osteoporosis, and impaired gait speed (44). In the current study, substantial weight loss (6 kg or more during the last 6 months, or 3 kg or more during the last month) was used as the main indicator for malnutrition. If all assessment criteria are applied according to the Short Nutritional Assessment Questionnaire 65+ (SNAQ65+), the association between malnutrition and falls might even be stronger.

This study has some limitations that need to be considered when interpreting the results. First, due to the cross-sectional study design, causality cannot be inferred. Further studies with multiple follow-up measures are needed to draw conclusions on the direction of the associations. In addition, including a larger sample of hospitalized older adults can generate more information regarding the factors associated with falls among this subpopulation. Second, participants in the community-dwelling sample were recruited by sending a letter to ask if they were willing to participate. This may have resulted in selection bias in which vulnerable participants are underrepresented. However, our sample also included hospitalized older adults with a relatively high average age and poorer health outcomes. Third, dichotomous outcome measures were used for falls, which may have resulted in loss of information. However, this simplification increases the understanding for practice. A strength of this study is that potential risk factors were explored from a multidimensional perspective, including demographic characteristics, health indicators, and lifestyle factors. Moreover, a diverse study population of hospitalized and community-dwelling participants was included.

The results of this study confirm the multi-factorial nature of falling and the complex interaction of risk factors. Findings of this study imply that future fall prevention programs could be tailored to subpopulations that are vulnerable, such as malnourished or lonely older adults. Additional research is needed to determine gender differences in the underlying causes and/or circumstances of falls and across age groups. Moreover, the results of this study may be useful for screening by (informal) caregivers, health care professionals and policymakers to identify older adults at risk of falls. Future research is needed to explore longitudinal associations and to comprehensively examine the (bi-) directional associations between risk factors and falls over time.

## 5. Conclusion

The current study fills the knowledge gap in comprehensive examination of the fall risk factors in a diverse older adult population. Female sex, education level, multimorbidity, a higher score on ADL limitations, loneliness and a high risk of malnutrition were associated with falling. More research, using longitudinal designs among a diverse and representative sample, is needed to confirm these findings. Accurate identification of high-risk groups and modifiable risk factors for falls is crucial for developing effective prevention programs tailored to the needs of hospitalized and community-dwelling older adults. It is recommended to further develop effective and feasible interventions to prevent falls among older adults and to contribute to their health and wellbeing.

## Data availability statement

The datasets presented in this article are not readily available because requests from third parties need to be reviewed first. Requests to access the datasets should be directed to h.raat@erasmusmc.nl.

## Ethics statement

The studies involving human participants were reviewed and approved by The Medical Ethics Committee of Erasmus MC University Medical Center (reference number: MEC-2016-559). The patients/participants provided their written informed consent to participate in this study.

## Author contributions

The study was conceptualized and designed by EB, AG, and HR. EB performed all statistical analyses and drafted the manuscript. ST and FM-R contributed to the data collection. EP and TA-B obtained funding for the APPCARE project. All authors contributed to the article and approved the submitted version.

## Funding

The authors received financial support for the research from the APPCARE project. APPCARE was funded by the European Commission, Grant Award Number: 664689.

## Conflict of interest

The authors declare that the research was conducted in the absence of any commercial or financial relationships that could be construed as a potential conflict of interest.

## Publisher’s note

All claims expressed in this article are solely those of the authors and do not necessarily represent those of their affiliated organizations, or those of the publisher, the editors and the reviewers. Any product that may be evaluated in this article, or claim that may be made by its manufacturer, is not guaranteed or endorsed by the publisher.
